# Resistance training program for fatigue management in the workplace: exercise protocol in a cluster randomized controlled trial

**DOI:** 10.1186/s12889-016-3872-5

**Published:** 2016-12-22

**Authors:** Hélio Gustavo Santos, Luciana Dias Chiavegato, Daniela Pereira Valentim, Patricia Rodrigues da Silva, Rosimeire Simprini Padula

**Affiliations:** 1 Masters and Doctoral Programs in Physical Therapy, Universidade Cidade de São Paulo, Rua Cesário Galeno 475, São Paulo, SP 03071-000 Brazil; 2 São Camilo University Center, Cachoeiro de Itapemirim, Espirito Santo Brazil; 3 Pulmonology Division, Universidade Federal de São Paulo, São Paulo, Brazil; 4 Departmento of Physical Therapy, Universidade Cidade de São Paulo, São Paulo, Brazil

**Keywords:** Fatigue, Resistance Exercise, Industrial workers, Physical Therapy

## Abstract

**Background:**

Fatigue is a multifactorial condition that leads to disease and loss in production, and it affects a large number of workers worldwide. This study aims to demonstrate a resistance exercise protocol that individuals will perform during the work schedule, and to evaluate the effectiveness of this exercises program for fatigue control.

**Methods/Design:**

This is a cluster randomized controlled trial with two arms and is assessor blinded. A total of 352 workers of both sexes, aged 18–65 years, from a medium-sized dairy plant were enrolled in this study. Participants will be recruited from 13 production sectors according to the eligibility criteria and will be randomized by clusters to either the Progressive Resistance Exercise (PRE) intervention group or the Compensatory Workplace Exercise (CWE) comparative group. A resistance exercise program will be implemented for both groups. The groups will receive instructions on self-management, breaks, adjustments to workstations, and the benefits of physical exercise. The PRE group will perform resistance exercises with gradual loads in an exercise room, and the CWE group will perform exercise at their workstations using elastic bands. The exercise sessions will be held 3 times a week for 20 min. The primary outcome measures will be symptoms of physical and mental fatigue, and muscular fatigue based on a one-repetition maximum (1RM). The secondary outcome measures will be level of physical activity, musculoskeletal symptoms, physical condition, perceived exposure, and productivity. The workers will be assessed at baseline and after a 4-month program. A linear mixed model will be applied on an intention-to-treat basis.

**Discussion:**

This intervention is expected to reduce symptoms of fatigue in the workers. The exercise program is indicating in the workplace, although there are few studies describing the effects of exercise on the control of fatigue in the workplace. Emphasis will be placed on adherence to the program, which may result in significant and clinically important reductions in fatigue. It is also expected that the findings of this study will contribute significantly to the decision-making capacity of professionals working in the field of occupational health.

**Trial registration:**

U.S. National Institutes of Health, ClinicalTrials.gov Identifier: NCT02172053. Date registered 19 June 2014.

## Background

Fatigue is a nonspecific symptom associated with chronic health problems and functional deterioration at work. It is complex and multidimensional and varies in intensity depending on the imposed overload [1–9]. Workplace fatigue is a common complaint that requires attention due to its high prevalence and its association with serious dysfunctions among workers [5]. It affects physical and mental health [3, 9], increasing the chance of accidents and musculoskeletal complaints [10], and reducing performance and productivity [4].

The symptoms of fatigue are due to individual characteristics [5, 6] as well as to work factors involving physical and mental demands, such as lack of planning with regard to work activities, rosters, and work shifts; environmental conditions; and standing for long periods [9]. Factors related to fatigue outside the workplace are sedentary behavior, lifestyle, and unhealthy diet, all of which build up over time [9]. Fatigue can be mental, due to prolonged periods of high cognitive demands along with the physical activity imposed by the daily load of highly physical jobs [7, 9]. Acute fatigue is a normal phenomenon in healthy workers and it is reversed after a period of rest [2]; however, chronic fatigue is more severe and often cannot be reversed simply by reducing workloads or resting [3, 6, 8]. The effects of fatigue on worker health and job performance can be short term or long term [2, 3, 6]. The short-term effects are reduced attention span, poor decision-making, reduced alertness, and poor control of emotions [3]. Fatigue can also increase the rate of mistakes, reduce reaction times, and elevate the likelihood of accidents and injuries [11]. The long-term effects are heart disease, diabetes, high blood pressure, gastrointestinal disorders, sleep loss, depression, and anxiety [2].

Fatigue contributes to the occurrence of musculoskeletal disorders, which represent a major problem for the health of workers worldwide [4, 5]. Workplace characteristics, repetitive tasks, static contractions, and inadequate posture are associated with the majority of structural disorders and the development of fatigue [10]. Various organizational aspects of work have been associated with a variety of adverse health effects, especially occupational disorders such as fatigue [10]. Thus, it becomes crucial to adopt measures for evaluating and managing these organic dysfunctions manifested as the development of fatigue [7]. Exercise programs have been widely used for fatigue management and pain relief, and to improve muscle strength, flexibility, and cardiovascular conditioning [12–15].

Exercise has been shown to have highly beneficial effects on physical and mental health [16], promoting significant changes to lifestyle and wellbeing [17]. It has a great impact on the health of all individuals, reducing mortality rates and increasing life expectancy [12, 13], as well as improving function in the musculoskeletal, blood, cardiopulmonary, immune, and nervous systems [12, 18]. Furthermore, it can reduce many of the risk factors for non-communicable chronic diseases (hypertension, cholesterol levels, diabetes), as well as percentage of body fat and body mass index [18, 19].

Exercise at the workplace has positive effects on the health of workers and is most effective when done in a group because there is more motivation thus increasing adherence to the program [20]. There is strong evidence of the effectiveness of strength training at the workplace for reducing musculoskeletal complaints in specific regions of the body [20–24]. A variety of strength training protocols are described in the literature, including protocols to decrease pain in the cervical, lumbar, and shoulder areas; low-intensity training; high-intensity training with concentric contractions; high-intensity training with isometric contractions; and highly intensive training (HIT) [20–22, 25]. However, there is consensus that resistance exercises are more effective [20, 25], and 20-min training sessions [20–25] 3 times a week for periods of 10 weeks or more [20, 23] reduce musculoskeletal complaints in the workplace. The protocol of heavy resistance exercise at the workplace includes exercise with higher intensity in eccentric and concentric contractions, using dumbbells, elastic bands, and exercises against gravity [20]. The effectiveness of medium- and long-term progressive resistance exercise programs with progressive loads for muscle strength gain and fatigue reduction has already been shown in the literature [18, 21], with most studies being cross-sectional in nature. In contrast, the small number of longitudinal studies that describe the benefits of resistance training for fatigue management in the workplace hinders decision-making regarding interventions for this population. Therefore, the advantage of this study is its randomized clinical trial design in the occupational context, with control of all of the variables that simulate the gym environment.

The hypothesis investigated in this study is that the physical load, high work demand, and absence of breaks imposed on production workers can contribute to an increase in the need for recovery due to increased symptoms of fatigue. It is understood that improving the workers’ physical conditioning is essential to managing the symptoms of fatigue at the workplace. To achieve that, resistance training with progressive loads is the most effective program.

Thus, our objective in this study is demonstrate a resistance exercise protocol to be performed at the workplace during the work schedule, and to describe the procedure that will be used to evaluate the effectiveness of this program in fatigue management for industrial workers.

## Methods

### Study design, approval, and registration

This is a cluster randomized controlled trial (RCT) two-arm (parallel group) with double blinded (investigator and assessor). The study protocol was approved by the Institutional Research Ethics Committee (Approval Number: 454709) according to the Helsinki Declaration as revised in 2013. Previously registered at ClinicalTrials.gov under protocol number NCT02172053. This protocol was reported according to SPIRIT guidelines.

### Setting and study sample

**Fig. 1:**
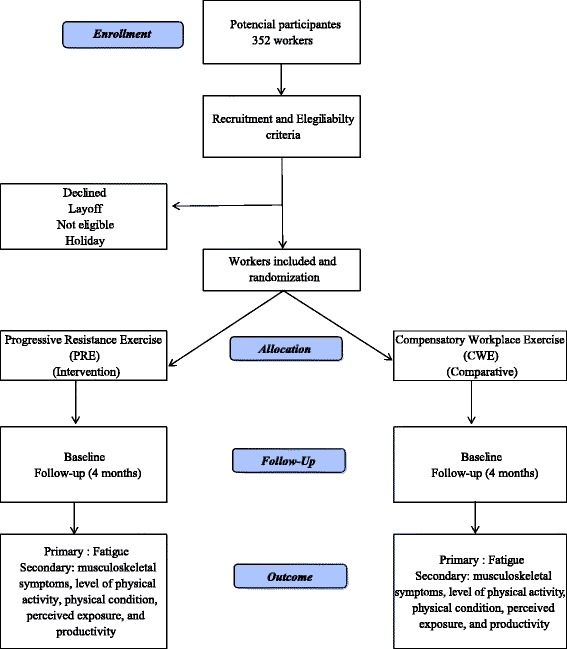
Flowchart of participants recruitment and study design

The study will include workers of both sexes, aged 18–65 years, recruited from production lines in a medium-sized dairy plant located in the state of Espírito Santo, Brazil. Workers exposed to moderate to high levels of biomechanical and cognitive demands who meet the eligibility criteria will be invited to participate. All information about the study design is in Fig. 1.

### Eligibility criteria

The dairy plant has 600 employees among its administrative and production sectors. The study will focus on the production sector, which enrolled 352 workers. The inclusion criteria will be as follows: permanent employment status, fixed work shifts, agreement to participate, and signing the informed consent form. The exclusion criteria will be temporary employment status, vacation, or sick leave to ensure group comparability, the feasibility of the interventions, and blinding.

### Randomization

Prior to clustering according to exposure level, 13 of the plant’s production sectors will be included in the study: Boilers, Processing, Receiving/Cooling/Standardization, Milk, Butter, Cheese, Milk Caramel, Yoghurt, UHT Plant, Milk Powder, Stock, and Warehouse. Allocation will be performed on Research Randomizer (https://www.randomizer.org/) by a researcher not involved with the data collection.

### Blinding

Due to the nature of the intervention, it is not possible to blind the workers and physical therapists who carry out the interventions. However, the researchers who will conduct the interviews and assessments will be blinded (double blinded). After the assessment, the blinding will be tested by having the researchers guess the type of intervention the worker received: Progressive Resistance Exercise (PRE) intervention group or the Compensatory Workplace Exercise (CWE) and write it down.

### Intervention protocols

Workers from both groups will receive initial instructions on health management (impact of fatigue, management of work demands, rest breaks, set up of workstations) and the importance of exercise to promote health and lifestyle changes. After the implementation of the exercise programs, these guidelines will be reinforced daily throughout the intervention period.

### Progressive Resistance Exercise (PRE) - intervention group

Workers allocated to this training program will perform light warm-up and stretching exercises followed by specific training with resistance and strength exercises. The training program of the PRE group will focus on muscle resistance, starting at 30% of the one-repetition maximum (1RM) [26]. The speed of the exercise will be moderate to allow control of the angle of movement. Load increase will be progressive according to each worker’s adaptability and physiological characteristics. Training will follow the principles of resistance training, starting with the adaptation to load phase (microcycle) and progressing to the load incorporation phase (mesocycle) and the training phase (macrocycle) [26]. The following muscle groups will be trained: elbow flexors, elbow extensors, trunk flexors, trunk extensors, knee flexors, knee extensors, thigh adductors, thigh abductors, and ankle dorsal and plantar flexors.

All exercises will be carried out in 3 sets of 10 repetitions with a 30-s interval between sets. The training will be held at the workplace in a room equipped with dumbbells, ankle weights, weight plates, and bars.

### Compensatory Workplace Exercise (CWE) – comparative group

Participants in this training program will have a light workout-involving warm-up and stretching exercises and resistance training with elastic bands in groups at their workstations. The protocol includes training of the following muscle groups: elbow flexors, elbow extensors, trunk flexors, trunk extensors, knee flexors, knee extensors, thigh adductors, thigh abductors, and ankle dorsal and plantar flexors. All exercises will be carried out in 3 sets of 10 repetitions with a 30-s interval between sets.

### Supervision and training schedule

Interventions will take place over a period of 4 months, beginning in August 2016. The outcomes will be assessed at baseline, and there will be a follow-up at the end of the intervention. Both groups of participants will train for 20 min, 3 times a week on alternate days. All interventions will be carried out during working hours at the workplace, totaling 1 h per week. All exercise protocols developed for this study are shown in Appendix I.

Ten experts will be involved in the research and will receive 12 h of training on the protocol and objectives of the study, regardless of their role (assessors, instructors, or lecturers). The researcher will supervise the training sessions at the workplace. Heart rate and blood pressure will be measured before every session. If any significant change that could hinder training is identified in any vital signs, the participant will be sent to the company’s outpatient clinic for assessment.

### Procedures

The workers’ biomechanical overload and occupational exposure levels were assessed using Quick Exposure Check (QEC) [27, 28]. The QEC assesses the following work-related biomechanical risk factors and exposure levels: frequency of movements and postures involving the spine and upper limbs; amount of weight handled; task completion time; manual strength; visual demand of the task; use of vibrating tools; work pace; and stress level. The total score of the instrument varies from 46 to 269 points, and the risk of exposure is classified into 4 categories: low (46–84 points), moderate (106–138 points), high (168–198 points), and very high (187–242 points) [27]. The levels of task complexity were classified as either easy or difficult according to task characteristics such as cognitive demands and learning time [29].

After analysis the demands in all productive sectors, these will be included in the study. The workers will be contacted and asked for their informed consent. Those who agree to participate will be evaluated and included based on the eligibility criteria. The participants will then be interviewed for demographic data collection (age, sex, education level, working hours, current role, working days per week, hours of work per week, etc.) and will be assessed using an individual form. Next, they will answer the questionnaires to evaluate the perception of need for recovery, musculoskeletal symptoms, and general health condition. Finally, they will undergo a battery of physical tests. Before the start of the intervention, all participants will attend a 30-min lecture on the importance of training (workplace exercise).

In the second phase of the project, the training programs with the intervention and comparative groups will be implemented. Events such as absences, complaints, expressions of satisfaction or discontent, and dropouts will be recorded in a logbook.

### Outcome measures

The primary outcome measures will be perception of fatigue and the secondary outcome measures will be musculoskeletal complaints and pain, quality of life, level of physical activity, physical condition, perceived exposure to risk factors, and productivity. The data collection instruments for these outcomes are described below. All outcomes will be evaluated at baseline and after 4 months (at the end program).

### Primary outcome measures

#### Perception of fatigue

The symptoms of fatigue attributed to work-related physical, organizational, and psychosocial demands and stress will be evaluated using the Need for Recovery Scale (Br-NFR) [30–32]. This Likert-type scale has 11 questions with 4 possible responses (0 = never; 1 = sometimes; 2 = often; and 3 = always). The answer “always” indicates an unfavorable situation and receives 3 points, except for item 4, which has a reversed score. The total score is obtained by adding all of the scores and converting them into a scale ranging from 0 (lowest) to 100 (maximum) by means of a simple rule of three [32]. In this case, the higher the score is, the greater the number of symptoms and the greater the need for recovery.

#### Muscular fatigue

Muscular fatigue will be evaluated using the 1RM test, which is the maximum amount of weight that can be lifted one time while performing a standardized exercise. The test will be completed when the individual 1RM reference value is found. The one-repetition maximum will be tested in the following muscle groups: biceps, triceps, deltoid, quadriceps femoris, hamstrings, and triceps surae, using an appropriate load for the individual’s fitness level.

### Secondary outcome measures

#### Musculoskeletal symptoms

The presence of musculoskeletal symptoms (pain, tingling, or numbness) will be assessed using the Nordic Musculoskeletal Questionnaire (NMQ) [33]. The respondents will answer simple yes/no questions related to musculoskeletal symptoms in the last 12 months and/or in the past 7 days, the occurrence of functional disability, and the need to seek assistance from health professionals due to the symptoms. Pain intensity will be evaluated using the Pain Numeric Rating Scale [34], an 11-point scale in which 0 means “no pain” and 10 means “the worst possible pain.”

#### Level of physical activity

The Baecke Physical Activity Questionnaire [35] will be used to assess the level of habitual physical activity (HPA) of the participants. It is a reminder tool, consisting of 16 questions covering three HPA scores for the previous 12 months: physical activity at work, sport during leisure time and other physical activities during leisure and locomotion. The score obtained at baseline will be used to classify individuals as sedentary or active, and the follow-ups will show any changes in physical activity levels over the intervention period.

#### Perceived risk

In this study, we will use the Job Factor Questionnaire to evaluate the workers’ perception of risk factors associated with the development of musculoskeletal complaints [36]. This instrument presents a descriptive list of 15 risk factors that are rated on a scale of 0 to 10 according to their contribution to the emergence of work-related musculoskeletal symptoms, with 0 indicating “no problem” and 10 indicating the “largest possible problem.”

#### Physical fitness assessment

1. Postural assessment - static assessment to identify postural changes with asymmetry that may affect training. This assessment will be carried out by direct observation and recorded on a specific form.
2. Vital signs - heart rate, respiratory rate, lung auscultation, blood pressure, and oxygen saturation. These signs will be assessed using a heart monitor (POLAR - RS800CX), a fingertip pulse oximeter, a stethoscope, and a sphygmomanometer. The aim is to monitor the individual before, during, and after exercise.
3. Body Mass Index (BMI) - a widely used parameter to estimate an individual’s health according to their weight and height. The World Health Organization (WHO) uses this index as an indicator of obesity levels in different countries. It is calculated by dividing the weight (kg) by the height squared (meters).
4. Waist-Hip Ratio (WHR) and Waist Circumference - WHR is an excellent way to identify the existence of increased risk for cardiovascular disease. Scientific studies have shown that a high concentration of abdominal fat (near the heart), without even considering the degree of obesity, is a risk factor for the development of heart disease [37]. The following equation will be used to measure WHR: waist measurement divided by hip measurement (W ÷ H). The higher the values are, the higher the risk. Results greater than or equal to 0.8 for women and 1.0 for men indicate a high risk for cardiovascular disease. Waist circumference is a measure that helps identify the people most likely to suffer from cardiovascular diseases [38] and it is as important as the BMI. A circumference greater than or equal to 94 cm in men and 80 cm in women is an indicator of a 3.25 times higher risk of developing heart disease [38]. A tape measure will be used to measure waist circumferences.
5. Body Fat Percentage - assessed with a body fat caliper commonly used in epidemiological research, outpatient clinics, doctor’s practices, and gyms. This apparatus features rulers that measure the fat in the skinfolds at different sites (triceps, biceps, pectoralis, subscapularis, midaxillary, suprailiac, abdomen, thigh, and calf). With these measurements, the professional can make a precise assessment of body composition and monitor the patient accordingly.
6. One-Repetition Maximum (1RM) - this test evaluates muscle strength. It measures the maximum amount of weight that an individual can lift in a single repetition. The 1RM will be tested in the following muscle groups: biceps, triceps, deltoid, quadriceps femoris, hamstrings, and triceps surae using an appropriate load for the individual’s fitness level. Dumbbells, weight plates, and other conventional weights will be used for this measurement [26].
7. Somatotype Rating - this assessment will identify the workers’ body type or physical classification. The terms endomorph (fat), mesomorph (muscular), and ectomorph (thin) will be used to describe the workers’ somatotype according to their weight, height, and body fat percentage at baseline [26].
8. Neck Circumference (NC). This measure is indicative of the level of obesity. A very large neck circumference may be related to increased risk of heart disease and metabolic disorders. A neck circumference ≥ 37 cm for men and ≥ 34 cm for women is equivalent to a BMI ≥ 25 kg/m^2^. A neck circumference ≥ 39.5 cm for men and ≥ 36.5 for women is equivalent to a BMI ≥ 30 kg/m^2^. A neck circumference of up to 37 cm in men and up to 34 cm in women indicates a normal BMI [39]

#### Productivity

The workers’ will answer a single question related to productivity at work during the follow up. This question is one of the items on the WHO Health and Work Performance Questionnaire (HPQ) [40] and asks the respondent to assign a score (0–10) to their work productivity over the previous 3 months.

#### Sample size

The sample size calculation is based on the difference detected in the Need for Recovery Scale (Br-NFR) [30, 31], that is, 20%. This difference was detected in the analysis of the average need for recovery observed over 7 working days and assessed at the beginning and the end of the shifts of 123 workers. Considering α = 0.05, a statistical power of 80%, and a sample loss of up to 15%, the sample size required per group is 86 workers (172 workers in total).

#### Statistical analysis

The data will be monitored by a committee not involved with data collection in order to avoid conflict of interest. A researcher will receive the encoded data and perform the statistical analysis. All data will be entered into the database twice, and the coding will be blinded. Descriptive statistics (frequencies, means, standard deviation, standard error, confidence interval) will be used to analyze the sociodemographic characteristics of the participants. The Shapiro-Wilk test will be used to assess the normality of the data. The chi-square test will be used to evaluate assessor blinding through a comparison between randomization codes and the assessors’ guesses. The difference between the groups and their respective confidence intervals will be calculated using a mixed linear model. The significance level will be 5%. The statistical program SPSS Statistics 24.0 will be used for all analyses, which will be performed on an intention-to-treat basis.

#### Ethical considerations

This study follows all ethical considerations set out in the Declaration of Helsinki. The study will present moderate risk because the participants will be exposed to muscular resistance exercises. This exposure will occur during the maximum load assessment to determine the training load percentage. The participants may experience changes in blood pressure, heart rate, and respiratory rate, according to their fitness level. To manage the risks, the research team will be ready to assist them should they perceive any changes and will refer them to the company’s medical department for attention if necessary. If the medical department subsequently releases the participant, he or she will be included in his or her allocated exercise program. All risks will be minimized by respecting the individual needs of each worker and always measuring their vital signs. The study results shall remain private and confidential. There are no conflicts of interest on the part of the authors and/or the company.

## Discussion

This study was designed to investigate the effectiveness of a resistance-training program for a group of workers at a medium-sized dairy plant to manage the symptoms of fatigue. Despite being a highly relevant topic, few studies have assessed the effect of resistance training at the workplace. We expect that this intervention with resistance training will have high adherence by the workers and will reduce the occurrence of fatigue symptoms. The program will bring many benefits to the participants, including health maintenance, reduced perception of fatigue, reversal of fatigue, reduction in complaints and pain, improved quality of life, higher productivity, improved mental health, and positive changes in lifestyle. We also expect that the results of this study will contribute significantly to the decision-making capacity of professionals working in the field of occupational health. We believe that both exercise protocols can be effective for fatigue reduction; however, we hypothesize that the PRE intervention protocol will be more effective.

### Trial status

Ongoing.

## Appendix I

### Resistance Exercise Protocol for Industrial Workers

#### Warm-Up stretches: intervention and comparative groups

Pre-intervention stretching to warm-up and prepare for exercise.

Stretching will involve cervical, upper/lower limb, and trunk muscles.

Seven warm-up/stretching exercises (two repetitions of 20 s each) for each muscle group, with attention to proper postural biomechanics and diaphragmatic breathing during stretching.

#### Starting movements

1. Stretching for posterior and anterior cervical muscles: Standing with hands on back of head, perform cervical flexion, then place hands on chin and perform cervical hyperextension.
2. Stretching for upper limb muscles: Standing with arm over chest and holding elbow with opposite hand, pull arm toward opposite shoulder. Then, standing with arm behind the head and holding elbow with opposite hand, perform elbow flexion and pull elbow down (right and left sides).
3. Stretching for the trunk: Perform trunk flexion with feet parallel and lower limbs fully extended.
4. Stretching for lower limbs: Standing upright, perform knee flexion while reaching behind and holding ankle, then dorsiflexion of feet with support (right and left side).

### Progressive Resistance Exercise (PRE) - intervention group

The exercises for the intervention group will include 3 series of 10 repetitions for each muscle group using dumbbells to increase muscle resistance, with a 30-s interval between series.

#### Intervention group: resistance exercises – moderate- and high-intensity resistance exercises using progressive loads with dumbbells:

1. Resistance exercises for biceps brachii: Standing with feet parallel, flex and extend elbows bilaterally at medium speed (3 series of 10 repetitions).
2. Resistance exercises for triceps brachii. Standing with feet parallel and shoulders in maximal flexion, flex and extend elbow unilaterally at medium speed (3 series of 10 repetitions; right and left sides).
3. Resistance exercises for shoulder muscles (deltoid, supraspinatus, and middle fibers of trapezius). Standing with feet parallel, perform shoulder abduction and adduction bilaterally at medium speed (3 series of 10 repetitions).
4. Resistance exercises for lower limb muscles – thigh and pelvic girdle (quadriceps femoral, gluteus, posterior thigh). Standing with lower limbs parallel, squat by flexing hip joint and knees bilaterally, at medium speed (3 series of 10 repetitions).
5. Resistance exercises for lower limb muscles – thigh and pelvic girdle (quadriceps femoral, gluteus, posterior thigh, abductors, and adductors). Standing with abducted and externally rotated lower limbs, squat by flexing hip joint and knees bilaterally at medium speed (3 series of 10 repetitions).
6. Resistance exercises for lower limb muscles – leg (triceps surae). In the standing position, perform bilateral plantar flexion at medium speed (3 series of 10 repetitions).

### Compensatory Workplace Exercise (CWE) - comparative group

The exercises for the Comparative Group will include 3 series of 10 repetitions for each muscle group with the use of elastic bands to increase muscle resistance, with a 30-s interval between series.

#### Control group: workplace exercise – Low - and moderate-intensity resistance exercises using elastic bands:

1. Resistance exercises for biceps brachii: Standing with lower limbs parallel and feet on the elastic band, hold the ends of the elastic band and flex/extend elbow bilaterally at medium speed (3 series of 10 repetitions).
2. Resistance exercises for triceps brachii: Standing with shoulders fully flexed and holding the ends of the elastic band, flex/extend elbow unilaterally at medium speed (3 series of 10 repetitions; right and left sides).
3. Resistance exercises for shoulder muscles (deltoid, supraspinatus, and middle fibers of trapezius). In the standing position, perform shoulder abduction and adduction bilaterally at medium speed (3 series of 10 repetitions).
4. Resistance exercises for lower limb muscles – thigh and pelvic girdle (quadriceps femoral, gluteus, posterior thigh). Standing with feet apart on the middle of the elastic band and holding the ends, perform bilateral hip joint and knee flexion at medium speed (3 series of 10 repetitions).
5. Resistance exercises for lower limb muscles – thigh and pelvic girdle (abductors and adductors). Standing with feet apart on the middle of the elastic band and holding the ends, perform unilateral hip joint abduction and adduction at medium speed (3 series of 10 repetitions; right and left sides).
6. Resistance exercises for lower limb muscles – leg (triceps surae). Standing with feet parallel, perform bilateral plantar flexion at medium speed (3 series of 10 repetitions).

## Abbreviations

1RM: 1 repetition maximum
BMI: Body Mass Index
Br-NFR: Brazilian - Need for Recovery Scale
CWE: Compensatory Workplace Exercise
HPA: Habitual physical activity
HPQ: Work Performance Questionnaire
NC: Neck Circumference
NMQ: Nordic Musculoskeletal Questionnaire
PRE: Progressive Resistance Exercise
QEC: Quick Exposure Check
RCT: Randomized controlled trial
WHO: World Health Organization
WHR: Waist-Hip Ratio
